# Nonvoluntary or Forced Sex Among Women, by Sexual Identity, Attraction, and Behavior — National Survey of Family Growth, United States, 2011–2017

**DOI:** 10.15585/mmwr.mm7038a3

**Published:** 2021-09-24

**Authors:** Grace S. Liu, Christopher R. Harper, Michelle M. Johns, Laura M. Mercer Kollar

**Affiliations:** ^1^Division of Violence Prevention, National Center for Injury Prevention and Control, CDC; ^2^Division of Adolescent and School Health, National Center for HIV/AIDS, Viral Hepatitis, STD, and TB Prevention, CDC.

Nonheterosexual (sexual minority) women report experiencing more sexual violence than heterosexual (sexual majority) women (*1*,*2*). Sexual minority women are often categorized as a collective whole, which fails to capture the nuances in sexual violence among subgroups of sexual minority women, such as bisexual and lesbian women (*3*). To estimate the prevalence of lifetime forced vaginal intercourse (forced sex) and of nonvoluntary first vaginal intercourse among women aged 18–44 years in the United States, CDC analyzed data from female respondents who were interviewed during 2011–2017 for the National Survey of Family Growth (NSFG); respondents were stratified by self-reported sexual identity, attraction, and behavior. Log-binomial regressions and analyses of variance (ANOVAs) were performed to compare experiences across each dimension of sexual orientation, controlling for demographic characteristics. Compared with sexual majority women,* prevalence of any male-perpetrated nonvoluntary first vaginal intercourse or forced sex (nonvoluntary or forced sex) was higher among women who identified as bisexual (36.1% versus 17.5%), reported attraction to the opposite and same sex (30.3% versus 15.8%), and reported sexual behavior with the opposite and same sex (35.7% versus 15.9%). These sexual minority women reported that their earliest experience of nonvoluntary or forced sex occurred at younger ages than did that of sexual majority women. Among women who were unsure of their sexual attraction, the prevalence of nonvoluntary first vaginal intercourse was also higher than among sexual majority women. These findings underscore the need for comprehensive prevention approaches tailored for sexual minority women and prevention of child sexual abuse, given the average ages at earliest nonvoluntary or forced sex experience among sexual minority women (range = 12.5–16.3 years). Additional research is needed into the circumstances of and norms or attitudes that influence perpetration of nonvoluntary or forced sex and broader sexual violence against sexual minority women. Prevention of nonvoluntary or forced sex victimization among sexual minority women will require comprehensive approaches to prevent sexual violence and child sexual abuse. Engaging sexual minority women in the development of sexual violence prevention efforts and research would help ensure that the experiences of sexual minority women across the spectrum are represented.

NSFG is a nationally representative, cross-sectional household survey conducted continuously by CDC’s National Center for Health Statistics (NCHS) that collects data on factors influencing reproductive health in the United States.^†^ Data from women aged 18–44 years^§^ in the three most recent releases (2011–2013, 2013–2015, and 2015–2017) were combined. Response rates for female participants ranged from 66.7% to 73.4%. To account for the multistage probability-based cluster sample design, 6-year weights provided by NSFG were applied during analysis using complex survey design functions in R statistical software (version 3.6.1; R Foundation) to reflect the female household population of the United States at the midpoint of data collection (July 2014). Sexual orientation, a multidimensional function of sexual identity, attraction, and behavior, was captured through measures of self-reported sexual identity^¶^ and attraction** at time of interview and lifetime sexual behavior^††^ (any sexual contact). Respondents aged ≥18 years were asked about experiences with nonvoluntary and forced sex: “Did you choose to have [first vaginal intercourse] of your own free will or not?” (nonvoluntary first vaginal intercourse), and “At any time in your life (besides the time you already reported), have you ever been forced by a male to have vaginal intercourse against your will?” (forced sex). Responses from both questions were used to create the collective measure of nonvoluntary or forced sex. Age at the earliest experience of nonvoluntary or forced sex was used in this analysis.

Estimated prevalence and associated 95% confidence intervals of any nonvoluntary or forced sex were calculated for respondents with nonmissing responses. Log-binomial regression models were used to generate prevalence ratios and test associations between each dimension of sexual orientation and nonvoluntary first vaginal intercourse or forced sex, adjusting for age, race and ethnicity, highest educational degree received, and poverty status.^§§^ ANOVAs were performed to compare mean age at first nonvoluntary or forced sex experience between women of different sexual orientations. Because of small cell sizes, results for nonvoluntary or forced sex among women with same-sex–only sexual behavior were omitted from the main findings in this report.^¶¶^This activity was reviewed by CDC and was conducted consistent with applicable federal law and CDC policy.***

The aggregated analytic sample included 14,309 female respondents.^†††^ Significant differences in demographic characteristics (i.e., race and ethnicity, educational attainment, and federal poverty level) were found (Table 1); 18.7% of respondents reported experiencing nonvoluntary or forced sex in their lifetime (Table 2). Lifetime prevalence of nonvoluntary or forced sex was higher among women identifying as bisexual (36.1%), those reporting attraction to the opposite and same sex (30.3%), and those reporting sexual contact with the opposite and same sex (35.7%) than among sexual majority women; among women not sure of their sexual attraction, prevalence was 28.9% (Table 2). Compared with sexual majority women, prevalence of nonvoluntary first vaginal intercourse was significantly higher among women identifying as lesbian (adjusted prevalence ratio [aPR] = 2.0) or bisexual (aPR = 2.4), reporting attraction to the same sex only (aPR = 2.2) or to the opposite and same sex (aPR = 1.9) or reporting sexual contact with the opposite and same sex (aPR = 1.9). Prevalence of nonvoluntary first vaginal intercourse was 3.7 times as high among women not sure of their sexual attraction as among women reporting opposite sex only attraction (Table 3).

**TABLE 1:** Sociodemographic characteristics of women aged 18–44 years (N = 14,309), by nonvoluntary or forced sex experience* — National Survey of Family Growth, United States, 2011–2017

Characteristic	Unweighted no. (weighted %)			p-value
	Overall (N = 14,309)	No nonvoluntary or forced sex (n = 11,402)	Any nonvoluntary or forced sex* (n = 2,907)	
**Age, mean (SD), yrs**	30.9 (7.7)	30.7 (7.7)	31.8 (7.7)	<0.001
**Race or ethnicity**				
White, non-Hispanic	6,824 (58.3)	5,351 (58.1)	1,473 (59.0)	<0.001
Black, non-Hispanic	3,193 (14.6)	2,472 (13.7)	721 (18.6)	
Hispanic	3,385 (20.1)	2,831 (20.9)	554 (16.7)	
Other	907 (7.1)	748 (7.3)	159 (5.8)	
**Highest degree received**				
No diploma or GED	1,875 (10.5)	1,457 (10.3)	418 (11.6)	<0.001
High school or equivalent	7,492 (50.2)	5,835 (48.5)	1,657 (57.6)	
Associate or bachelor’s	3,668 (28.7)	3,009 (29.6)	659 (24.5)	
Master’s or higher	1,274 (10.6)	1,101 (11.5)	173 (6.4)	
**Federal poverty level**				
≤130%	5,641 (32.9)	4,247 (31.1)	1,394 (40.6)	<0.001
>130%	8,668 (67.1)	7,155 (68.9)	1,513 (59.4)	

**Abbreviations:** GED = general education development certificate; SD = standard deviation.

* Nonvoluntary or forced sex experience was measured using responses to two questions: whether first vaginal intercourse was “voluntary or not voluntary, that is, did you choose to have sex of your own free will or not?” and “At any time in your life (besides the time you already reported), have you ever been forced by a male to have vaginal intercourse against your will?”

**TABLE 2:** Estimated prevalence of nonvoluntary or forced sex* among women aged 18–44 years (N = 14,309), by sexual orientation — National Survey of Family Growth, United States, 2011–2017

Sexual orientation	Unweighted no.	Any nonvoluntary or forced sex*		
		Unweighted no.	Weighted % (95% CI)	p-value^†^
**Total**	**14,309**	**2,907**	**18.7 (17.7–19.6)**	**N/A**
**Sexual identity^§^**				
Heterosexual or straight (sexual majority)	12,843	2,425	17.5 (16.5–18.4)	**<0.001**
Lesbian or gay	295	73	18.2 (12.7–24.8)	
Bisexual	991	362	36.1 (31.8–40.5)	
**Sexual attraction^¶^**				
Opposite sex only (sexual majority)	11,141	1,934	15.8 (14.8–16.9)	<0.001
Same sex only	194	51	19.8 (12.7–28.3)	
Opposite and same sex	2,740	850	30.3 (27.7–32.9)	
Not sure	213	67	28.9 (21.3–37.4)	
Lifetime sexual behavior**				
Opposite sex only (sexual majority)	10,801	1,854	15.9 (14.9–16.9)	<0.001
Same sex only^††^	—	—	—	
Opposite and same sex	2,715	1,031	35.7 (33.1–38.4)	
No sexual behavior	667	9	1.8 (0.4–5.1)^§§^	

**Abbreviations:** CI = confidence interval; N/A = not applicable.

* Nonvoluntary or forced sex experience was measured using responses to two questions: whether first vaginal intercourse was “voluntary or not voluntary, that is, did you choose to have sex of your own free will or not?” and “At any time in your life (besides the time you already reported), have you ever been forced by a male to have vaginal intercourse against your will?”

^†^ Comparisons within subgroups were evaluated on weighted prevalence estimates via chi-square tests. Statistical significance was evaluated at a threshold of α = 0.05.

^§^ Sexual identity was assessed by asking the respondents whether they thought of themselves (at time of the interview) as heterosexual or straight; homosexual, gay, or lesbian; or bisexual.

^¶^ Sexual attraction to other persons (at time of the interview) was assessed through self-reports of being only attracted to the opposite sex; mostly attracted to the opposite sex; equally attracted to the opposite and same sex; mostly attracted to the same sex; only attracted to the same sex; or not sure. Respondents who reported being mostly attracted to the opposite, equally attracted to the opposite and same sex, or mostly attracted to the same sex were coded as being attracted to both the opposite and same sex.

** Lifetime sexual behavior was measured using experiences of oral, vaginal, or anal sex with male or female partners at any time in the respondent's life. Based on responses to two separate survey questions regarding sexual experiences with male partners and female partners, a composite sexual behavior variable was created with four categories: opposite sex only, same sex only, opposite and same sex, and no sexual behavior.

^††^ Because of small cell sizes for reported nonvoluntary or forced sex among women reporting same-sex–only sexual behavior and ambiguity regarding interpretation, analysis results for this sexual behavior group have been omitted from main findings.

^§§^ Estimates for women reporting no sexual behavior with male partners are likely a reflection of the distinction between forced sex as an act of violence rather than a sexual experience.

**TABLE 3:** Prevalence ratios of experiences of nonvoluntary or forced sex among women aged 18–44 years (N = 14,309), by sexual orientation — National Survey of Family Growth, United States, 2011–2017

Characteristic	Adjusted PR* (95% CI)^†^			Mean (SE)^§^
	Nonvoluntary first vaginal intercourse^¶^	Forced sex**	Any nonvoluntary or forced sex^††^	Age at earliest occurrence of nonvoluntary or forced sex, yrs
**Sexual identity^§§^**				
Heterosexual or straight (sexual majority)	Ref	Ref	Ref	17.0 (0.2)
Lesbian or gay	2.0 (1.3–3.1)^¶¶^	1.1 (0.7–1.6)	1.0 (0.7–1.4)	12.7 (1.1)***
Bisexual	2.4 (1.8–3.2)***	2.0 (1.8–2.4)***	2.1 (1.8–2.3)***	15.7 (0.4)^¶¶^
**Sexual attraction^†††^**				
Opposite sex only (sexual majority)	Ref	Ref	Ref	17.1 (0.2)
Same sex only	2.2 (1.4–3.5)^¶¶^	1.2 (0.8–2.0)	1.3 (0.8–1.8)	12.5 (1.3)***
Opposite and same sex	1.9 (1.5–2.4)***	2.1 (1.9–2.4)***	2.0 (1.8–2.2)***	16.3 (0.3)^¶¶^
Not sure	3.7 (2.6–5.3)***	0.9 (0.6–1.5)	1.7 (1.2–2.2)***	17.1 (1.2)
**Lifetime sexual behavior^§§§^**				
Opposite sex only (sexual majority)	Ref	Ref	Ref	17.3 (0.2)
Same sex only^¶¶¶^	—	—	—	—
Opposite and same sex	1.7 (1.4–2.1)***	2.5 (2.2–2.7)***	2.2 (2.0–2.4)***	16.0 (0.3)***
No sexual behavior	N/A	0.2 (0.1–0.6)^¶¶^	0.1 (0.0–0.5)^¶¶^	9.9 (2.1)***

**Abbreviations:** ANOVA = analysis of variance; CI = confidence interval; GED = general education development certificate; N/A= not applicable; PR = prevalence ratio; Ref = referent group; SE = standard error.

* Adjusted PRs were controlled for age (years), at the time of interview race (non-Hispanic White, non-Hispanic Black, Hispanic, or other), highest degree received (no diploma or GED, high school diploma or GED, associate or bachelor’s degree, or master’s degree or higher), and poverty status (at or below or above federal poverty level).

† Comparisons within subgroups were evaluated on weighted prevalence estimates via log-binomial regressions used to calculate a prevalence ratio, 95% CI, and p-value (not shown). Statistical significance was evaluated at a threshold of α = 0.05.

^§^ Comparisons within subgroups were evaluated on weighted means via one-way ANOVA used to calculate a standard error and p-value. Statistical significance was evaluated at a threshold of α = 0.05.

^¶^ Nonvoluntary first vaginal intercourse was measured using responses to the question of whether first vaginal intercourse was “voluntary or not voluntary, that is, did you choose to have sex of your own free will or not?”

** Forced sex was measured using the survey question, “At any time in your life (besides the time you already reported), have you ever been forced by a male to have vaginal intercourse against your will?”

^††^ Nonvoluntary or forced sex experience used responses from both survey questions regarding nonvoluntary first vaginal intercourse and forced sex.

^§§^ Sexual identity was assessed by asking the respondents whether they thought of themselves (at time of the interview) as heterosexual or straight; homosexual, gay, or lesbian; or bisexual.

^¶¶^ P-value is statistically significant (p≤0.01).

*** P-value is statistically significant (p≤0.001).

^†††^ Sexual attraction to other persons (at time of the interview) was assessed through self-reports of being only attracted to the opposite sex, mostly attracted to the opposite sex, equally attracted to the opposite and same sex, mostly attracted to the same sex, only attracted to the same sex, or not sure. Respondents who reported being mostly attracted to the opposite, equally attracted to the opposite and same sex, or mostly attracted to the same sex were coded as being attracted to both the opposite and same sex.

^§§§^ Lifetime sexual behavior was measured using experiences of oral, vaginal, or anal sex with male or female partners at any time in the respondent's life. Based on responses to two separate survey questions regarding sexual experiences with male partners and female partners, a composite sexual behavior variable was created with four categories: opposite sex only, same sex only, opposite and same sex, and no sexual behavior.

^¶¶¶^ Because of small cell sizes for reported nonvoluntary or forced sex among women reporting same-sex–only sexual behavior and ambiguity regarding interpretation, analysis results for this sexual behavior group have been omitted from main findings.

Compared with sexual majority women, the prevalence of forced sex was significantly higher among women who identified as bisexual (aPR = 2.0), reported attraction to the opposite and same sex (aPR = 2.1), or reported sexual behavior with the opposite and same sex (aPR = 2.5) (Table 3). Prevalence of overall nonvoluntary or forced sex was approximately twice as high among women identifying as bisexual, reporting attraction to the opposite and same sex, and reporting sexual contact with the opposite and same sex as among sexual majority women and was significantly higher among women not sure of their sexual attraction than among those attracted only to the opposite sex (aPR = 1.7). Average age of earliest experience of nonvoluntary or forced sex was significantly younger among all sexual minority women (except women not sure of their sexual attraction) than among sexual majority women (ranges = 12.5–16.3 versus 17.0–17.3 years). Average age of first nonvoluntary or forced sex experience was youngest among women reporting no sexual behavior with males (9.9 years).^§§§^

## Discussion

These results are consistent with past findings that sexual minority women experience higher risk for nonvoluntary or forced sex than do sexual majority women, and that sexual minority women experience initial nonvoluntary or forced sex at younger ages than sexual majority women (*1*,*2*). These findings extend past research to demonstrate differences in prevalence of nonvoluntary or forced sex among subgroups of women along the spectrum of minority sexual identity, attraction, and behavior. A prominent explanation for the higher prevalence of violence experienced by sexual minority women is sexual minority stress theory, which hypothesizes that chronic stigma and discrimination contribute to the marginalization of sexual minorities (*4*); however, additional research is needed to understand the link between the early age of onset of violence given that some sexual minority women might not have identified as such when the violence occurred. Restrictive and harmful social norms regarding gender and sexuality might explain perpetration of violence against sexual and gender minorities (*5*). Bisexual women represent a unique subpopulation of sexual minority women who might further experience discrimination not experienced by their homosexual or lesbian peers (*6*). Rape myths portraying bisexual women as promiscuous and confused about their sexuality could increase perpetration against these women and explain their elevated risk for sexual violence victimization (*6*). Understanding harmful attitudes toward bisexual women in relation to sexual victimization and perpetration might help explain this higher prevalence ratio for bisexual women. Further research is needed to understand the social mechanisms, including circumstances of nonvoluntary or forced sex and norms and attitudes about sex and sexuality, that underlie differences in nonvoluntary or forced sex experiences among sexual minority women and bisexual women specifically (*7*).

Harmful attitudes and norms might also partly explain early occurrences of nonvoluntary or forced sex and high prevalence of nonvoluntary first vaginal intercourse among sexual minority women (*6*). By measuring sexual orientation in a multidimensional way, this study demonstrates the high prevalence of lifetime nonvoluntary or forced sex, and nonvoluntary first vaginal intercourse in particular, among women who are unsure of their sexual attraction. Disproportionate risk for nonvoluntary or forced sex, and potentially sexual violence more broadly, accompany minority sexual identity, attraction, and behavior. Approaching sexual orientation as a function of sexual identity, attraction, and behavior is critical to fully understanding the disparities in female sexual victimization, associations between sexuality and violence victimization and perpetration, and the early age at which sexual minority women first experience nonvoluntary or forced sex (*7*).

The findings in this report are subject to at least four limitations. First, gender identity (i.e., a person’s internalization of their own gender) or expression (i.e., a person’s outward presentation of their gender), which can further exacerbate risks of sexual violence associated with sexual identity (*2*), is not captured in NSFG. Sociodemographic characteristics were also accounted for but not evaluated in depth. Additional research is needed to understand how intersecting social identities of gender identity, race/ethnicity, and sexual minority status might interact in complex ways that could affect persons’ disproportionate experiences of nonvoluntary or forced sex (*8*). Second, this survey does not capture female-perpetrated nonvoluntary or forced sex, other types of penetration, or nonpenetration forms of sexual violence (e.g., attempted rape or sexual harassment), nor does it account for underreporting of nonvoluntary or forced sex (*9*). Third, cross-sectional surveys have the potential for recall bias and cannot confirm whether observed associations are causal. Longitudinal studies are needed to further understand experiences of sexual minority women across their lifetime, including the circumstances preceding nonvoluntary or forced sex. Finally, estimates of experience with nonvoluntary or forced sex among women reporting no sexual behavior with male partners are likely a reflection of the distinction between forced sex as an act of violence rather than a sexual experience (*10*). Inability to determine whether this distinction was consistent among all responses is a limitation of the sexual behavior measure; this inability highlights a need for expanded health research to more accurately and comprehensively capture the impact experienced by all subgroups of sexual minority women. Despite these limitations, these analyses provide nationally representative estimates of nonvoluntary or forced sex among sexual minority women, stratified by sexual identity, attraction, and behavior, in the United States.

Given the higher prevalence of nonvoluntary or forced sex associated with minority sexual identity, attraction, and behavior, comprehensive efforts to prevent victimization of sexual minority women are warranted. These findings highlight the need to engage sexual minority women in the development of targeted primary prevention efforts. Comprehensive efforts based on best available evidence, including emphasizing approaches to changing gender and sexuality norms and attitudes, improving bystander behaviors, empowering sexual minority women and girls, and creating protective environments, could be enhanced to address perpetration of nonvoluntary or forced sex and victimization among sexual minority women (*7*). The finding that sexual minority women experienced nonvoluntary or forced sex at younger ages than sexual majority women might also suggest a history of more frequent adverse childhood experiences and an increased risk for revictimization later in life (*5*). Therefore, in addition to expanding sexual assault measures in existing surveys, cohort studies are needed to better understand the marginalization and experiences of sexual minority women, including across intersecting identities (e.g., race and ethnicity, education level, and socioeconomic status) (*8*). Findings can guide tailoring of primary prevention efforts for sexual violence and adverse childhood experiences, such as child sexual abuse and teen dating violence. Sex education curricula that are inclusive of sexual and gender minority experiences might also promote healthy sexuality and safe intimate relationship skills that help sexual minority women (and all other sexual and gender minority persons) stay safe and healthy (*7*).

**Summary**

**What is already known about this topic?**

Sexual minority women are more likely to experience sexual violence than sexual majority women.

**What is added by this report?**

Among women aged 18-44 years surveyed during 2011–2017, lifetime prevalence of nonvoluntary or forced sex was highest among bisexual women (36.1%). Compared with sexual majority women, nonvoluntary first vaginal intercourse was more prevalent among sexual minority women; first experience of nonvoluntary or forced sex occurred at younger ages.

**What are the implications for public health practice?**

Prevention of nonvoluntary or forced sex victimization among sexual minority women will require comprehensive approaches to prevent sexual violence and child sexual abuse. Engaging sexual minority women across the spectrum in primary prevention efforts could help ensure intervention effectiveness.

## Footnotes

* Sexual majority refers to those whose sexual orientation aligns with the presumed majority of the population. The term is used here to include respondents who identified as being heterosexual or straight, reported attraction to the opposite sex only, or reported sexual behavior with the opposite sex only.

†
https://www.cdc.gov/nchs/nsfg/index.htm

§ From 2011 to 2015, NSFG was conducted among females aged 15–44 years. This age range was expanded in 2015 to include women aged 45–49 years. To maintain a consistent age range and apply NCHS-provided weights, respondents aged 45–49 years interviewed during 2015–2017 were excluded in analysis. Respondents aged <18 years were not asked questions relating to nonvoluntary or forced sex.

¶ Sexual identity was assessed by asking the respondents whether they thought of themselves (at the time of the interview) as heterosexual or straight; homosexual, gay, or lesbian; or bisexual.

** Sexual attraction to other persons (at time of the interview) was assessed through self-reports of being only attracted to the opposite sex; mostly attracted to the opposite sex; equally attracted to the opposite and same sex; mostly attracted to the same sex; only attracted to the same sex; or not sure. Respondents who reported being mostly attracted to the opposite sex, equally attracted to the opposite and same sex, or mostly attracted to the same sex were coded as being attracted to both the opposite and same sex.

†† Lifetime sexual behavior was measured using experiences of oral, vaginal, or anal sex with male or female partners at any time in the respondent's life. Based on responses to two separate survey questions about sexual experiences with male partners and female partners, a composite sexual behavior variable was created with four categories: opposite sex only, same sex only, opposite and same sex, and no sexual behavior.

§§ Sociodemographic characteristics included in the analysis were age at the time of interview, race and ethnicity (non-Hispanic White, non-Hispanic Black, Hispanic, or other), highest degree received (no diploma or general education development certificate [GED], high school diploma or GED, associate or bachelor’s degree, or master’s degree or higher), and poverty status (at or below or above 130% of the federal poverty level).

¶¶ Among the 97 persons reporting a history of same-sex–only sexual behavior, lifetime prevalence of nonvoluntary or forced sex was 3.7% (5; 95% confidence interval [CI] = 0.9–9.7). Compared with women reporting sexual behavior with the opposite sex only, among women reporting sexual history with the same sex only the prevalence of forced sex (aPR = 0.3; 95% CI = 0.1–1.0) and lifetime nonvoluntary or forced sex (aPR = 0.3; 95% CI = 0.1–0.8) were significantly lower. Average age of first experience of nonvoluntary or forced sex was significantly younger among those with same-sex–only sexual behavior than among those with opposite sex only sexual behavior (13.7 versus 17.3 years).

*** 45 C.F.R. part 46.102(l)(2); 21 C.F.R. part 56; 42 U.S.C. Sect.241(d); 5 U.S.C. Sect.552a; 44 U.S.C. Sect.3501 et seq.

††† Among 16,954 female respondents from the 2011–2017 surveys, 11.2% (1,893) were missing data on nonvoluntary or forced sex, 0.6% (105) had no data for any sexual orientation components, and 4.5% (763) were missing survey weights. Altogether, 2,645 respondents were excluded from analysis because of incomplete data.

§§§ Reports of nonvoluntary or forced sex among women who report no history of sexual behavior with males are likely due to a distinction between forced sex as an act of violence rather than a sexual experience.
